# HIV Care Continuum and Preexposure Prophylaxis Program in Federal Bureau of Prisons, United States

**DOI:** 10.3201/eid3013.230799

**Published:** 2024-04

**Authors:** Xiao Hong Huang, Elizabeth Thompson, Tami Rodriguez

**Affiliations:** US Public Health Service, Federal Bureau of Prisons, Washington, DC, USA

**Keywords:** HIV infections, preexposure prophylaxis, pharmacists, prisons, correctional facilities, viruses, HIV/AIDS, United States

## Abstract

In 2019, the US Department of Health and Human Services launched the Ending the HIV Epidemic in the US initiative (EHE) with the goal of reducing new HIV infections by 90% by 2030. This initiative identifies 4 pillars (diagnose, treat, prevent, and respond) to address the HIV epidemic in the United States. To advance the EHE goals, the Federal Bureau of Prisons (FBOP) has implemented interventions at all points of the HIV care continuum. The FBOP has addressed the EHE pillar of prevention through implementing preexposure prophylaxis, developing a strategy to decrease the risk of new HIV infection, and providing guidance to FBOP healthcare providers. This article describes the implementation of programs to improve the HIV care continuum and end the epidemic of HIV within the FBOP including a review of methodology to implement an HIV preexposure prophylaxis program.

In 2020, the Centers for Disease Control and Prevention (CDC) reported 30,635 new HIV diagnoses in the United States (*1*) and estimated that, for 13% of persons living with HIV (PLWH) in the United States, the HIV infection has not been diagnosed (*2*). In 2019, the US Department of Health and Human Services launched the Ending the HIV Epidemic in the United States initiative (EHE); the goal of EHE is to reduce new HIV infections by 90% by 2030. When used together, the 4 pillars of this initiative—diagnosis, treatment, prevention, and response—can end the HIV epidemic in the United States (*3*).

The Federal Bureau of Prisons (FBOP) supports the 4 pillars of EHE through HIV screening, offering antiretroviral treatment to all PLWH, effectively treating PLWH, and implementing an HIV preexposure prophylaxis (PrEP) program. When taken as prescribed, PrEP reduces the risk for new HIV infections through sexual exposure by 99% and risk for new infection through intravenous drug use by 74% (*4*); however, PrEP is still highly underused (*5*). CDC recommends PrEP for persons who are HIV negative and might be at risk for HIV infection, including those who have had anal or vaginal sex in the previous 6 months and have a sexual partner with HIV, have not consistently used a condom, or have had a diagnosis of a sexually transmitted infection (STI) in the previous 6 months (*5*). Persons with a history of incarceration have higher rates of many of those risk factors compared with the general public (*6*–*8*), making PrEP a key intervention to reduce the risk for HIV infection after release from custody. In this article, we describe the FBOP HIV testing and treatment program and explore the implementation of the FBOP PrEP program.

## FBOP HIV Program Description

### HIV Testing and Treatment Program, 2004–2023

FBOP HIV Clinical Practice Guidelines were established to treat PLWH. Since 2016, FBOP guidelines have recommended an opt-out strategy for HIV screening, in which all adults in custody (AICs) are informed that an HIV test will be performed as part of the standard laboratory screening upon entry into custody. If the AIC choses to opt-out of voluntary testing, a refusal is documented. Current guidelines recommend initiating treatment as soon as the patient is willing and able to start and provides scenarios when rapid initiation (i.e., same day as diagnosis) might be indicated (*9*). In December 2004, FBOP created an HIV Clinical Pharmacist Consultants program to enhance patient management. Currently, 15 pharmacist consultants with specialized HIV training assist with managing all PLWH in FBOP custody, including reviewing laboratory findings, clinical encounters, and prescription profiles to ensure appropriate HIV care. Consultants also serve as a resource for providers seeking expert consultation in HIV management.

### PrEP Program, 2021–2023

Although rates of new HIV infections during incarceration are low (*10*), >95% of AICs will return to their communities (*11*), where they might engage in behaviors that place them at risk for HIV infection, including intravenous drug use and unprotected sex (*6*–*8*). Recognizing the opportunity to decrease the risk for HIV transmission, FBOP updated its HIV Clinical Practice Guidelines in April 2021 to include guidance for oral PrEP (*9*).

Under the FBOP PrEP program, providers can identify patients with HIV risk factors or patients may self-refer for evaluation for PrEP. FBOP guidance identifies the following risk factors that might indicate an HIV-negative patient is at high risk for HIV infection upon release: vaginal or anal sex 6 months before or at any time during incarceration and partner with HIV, inconsistent use of condoms with partner(s) of unknown HIV status or at high risk for HIV, sex while using drugs, and/or >1 sex partner; sexually transmitted infection 6 months before or at any time during incarceration; shared needles for intravenous drug use 6 months before or at any time during incarceration, or might engage in intravenous drug use upon release. Working under a collaborative practice agreement, pharmacists can also perform patient assessments and prescribe PrEP. Although obtaining a detailed sexual activity and drug use history is a major part of the assessment, patients might be hesitant to disclose high-risk behaviors because of fear of discipline or stigma. Consequently, and consistent with CDC PrEP guidelines (*5*), confirmed high-risk behavior is not a requirement to provide PrEP to any releasing AIC who requests it.

To ensure adequate time to assess response to treatment and coordinate continuity of care before release, PrEP is initiated ≈30 days before release from custody, and a social worker creates a customized release plan to include scheduling a follow-up appointment for the patient in the community. Patients are also given a Release Resources for PrEP handout to assist with obtaining PrEP access should they be unable to complete their scheduled appointment. Upon release, a 90-day supply of PrEP medication is sent with the patient to allow ample time for linkage to care.

### Promoting the PrEP Program within FBOP

FBOP uses a multi-modal strategy to educate providers about the PrEP guidance and to encourage uptake among AICs. HIV Clinical Pharmacist Consultants developed a webinar for healthcare providers discussing harm-reduction strategies including PrEP. To educate AICs, HIV Clinical Pharmacists developed a PrEP Fact Sheet reviewing the basics of how PrEP works, how to start PrEP within the FBOP, and how to access medication. In addition, Clinical Pharmacists developed a bulletin that was posted nationally through the FBOP internal computer system available to all AICs in both English and Spanish. This bulletin informs AICs that PrEP is available for any person who is at risk for HIV infection and will soon be releasing to the community and encourages self-referral to Health Services providers for more information. The bulletin also states a 90-day supply of medication will be sent upon release and provides information about programs that might help pay for PrEP in the community.

Individual FBOP institutions have also developed strategies to increase PrEP uptake. For example, the United States Medical Center for Federal Prisoners in Springfield, Missouri, developed a pharmacist-led Harm Reduction Clinic. This clinic began in 2021 and initially focused on providing nasal naloxone to reduce overdose deaths after release from custody. In June 2022, the clinic was expanded to offer PrEP as well. To identify potentially eligible patients, every month the clinic generates a roster of AICs due to be released from custody within 90 days and schedules patients for an appointment with a pharmacist. Providers can also refer patients to the clinic, and self-referrals are encouraged. If a patient with a release date >90 days in the future is referred to the clinic, they receive harm reduction education and are rescheduled for evaluation for PrEP 90 days before their projected release date.

During the Harm Reduction clinic, pharmacists and patients discuss HIV risk factors, prevention methods, and resources, as well as information about what PrEP is, PrEP indications, and possible and anticipated side effects. PLWH are provided education about promoting PrEP use for existing or future HIV-negative partners. Pharmacists also perform medication reconciliation and discuss the patient’s overall health conditions to improve health literacy and continuity of care upon reentry. If PrEP is indicated, the pharmacist will order appropriate laboratory tests, provide monitoring and follow-up with the patient after initiation, and assist the patient with continuity of care coordination with a FBOP social worker. To ensure all necessary components of a harm reduction visit are included and to standardize the workflow and documentation process, the HIV Clinical Pharmacists developed a notes template for visits.

## Methods

We used the FBOP electronic medical record to determine the number and percentage of persons who entered custody since the beginning of the PrEP program (April 1, 2021–March 25, 2023) who reported HIV risk factors (including potential indications for PrEP) during the intake process. In addition, we used electronic medical records to calculate the FBOP HIV care continuum for the FBOP population as of June 2, 2023. HIV care continuum steps include the number of AICs who have been offered >1 HIV test during their incarceration, the number who had a positive test result, the number who are on treatment, and the number who have an undetectable viral load.

## Results

### FBOP Population and HIV Risk Factors

During April 1, 2021–March 25, 2023, a total of 303,817 intakes were completed for persons who newly entered FBOP custody or transferred between FBOP facilities. The most common self-reported HIV risk factor was infrequent condom use (146,665 [48.3%]), followed by never using a condom (111,592 [36.7%]) (Table).

**Table:** Prevalence of self-reported HIV risk factors during intake screening in study of HIV care and preexposure prophylaxis program in the Federal Bureau of Prisons, April 1, 2021–May 25, 2023*

Risk factors†	No. (%) reporting a risk behavior
History of STI‡	40,139 (13.2)
Intravenous drug use	20,287 (6.7)
IV drug use with needles	7,693 (2.5)
Sexual risk factors	259,843 (85.5)
Condom use	
Sometimes	146,665 (48.3)
Never	111,592 (36.7)
Sexual contact with HIV-positive person	1,586 (0.5)

*Total number of intakes was 303,817. Intake screening is performed each time an adult in custody enters a new facility. Because persons in Federal Bureau of Prisons custody often move between facilities, an intake might have been completed for the same patient >1 time during this period. IV, intravenous; STI, sexually transmitted infection. †Federal Bureau of Prisons guidance identifies risk factors that might indicate an HIV-negative patient is at high risk for HIV infection upon release, such as vaginal or anal sex 6 months before or at any time during incarceration and HIV-positive partner, inconsistent use of condoms with partner(s) of unknown HIV status or at high risk for HIV, sex while using drugs, >1 sex partner, STI diagnosis 6 months before or at any time during incarceration, and shared needles for IV drug use 6 months before or at any time during incarceration or might engage in IV drug use upon release. ‡Includes syphilis, genital warts, chlamydia, gonorrhea, and herpes.

### HIV Care Continuum

An evaluation of FBOP electronical medical record data for AICs in FBOP custody for >90 days showed that as of June 2, 2023, there were 139,789 AICs. Of those, 117,950 (89.6%) had been offered >1 HIV test. Of those, 1,208 (1.0.%) had a positive test result, 100% of PLWH had been offered antiretroviral treatment, and 1,199 (99.3%) had initiated treatment. As of the end of the study period, 1,110 patients had been on treatment >90 days; of those, 1,060 (95.5%) had an undetectable viral load (Figure).

**Figure:**
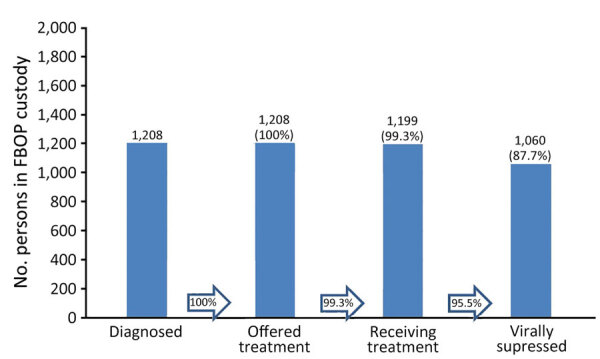
Number and percentage of adults in custody of the US FBOP for >90 days as of June 2, 2023, who had been diagnosed with HIV, offered treatment, accepted treatment, or were virally suppressed. Virally suppressed was defined as CD4 <200 copies/mL on the most recent test. FBOP, Federal Bureau of Prisons.

### Preliminary Data for PrEP Initiation

In 2022, a total of 28 patients from 24 institutions initiated PrEP within 90 days of release from FBOP custody. As of June 2023, a total of 41 patients from 29 institutions had initiated PrEP. To enable further analysis, the FBOP is developing a dashboard to include the number of AICs screened for PrEP eligibility, the number offered PrEP, and the percentage who accepted.

## Discussion

The HIV care continuum among AICs has improved substantially over the past 20 years; in 2003, only 32% of PLWH were virally suppressed (*12*), whereas 96% were virally suppressed in 2023. In addition, AICs now have access to PrEP to prevent HIV infection after release from custody.

Advances in HIV care within the FBOP coincide with the implementation of the FBOP Clinical Pharmacist Consultant Program. Given their extensive training and expertise in medication management, pharmacists are well equipped to manage HIV and PrEP. An evaluation of pharmacy-based initiatives to increase PrEP use in the community has shown that patients supported pharmacist-based PrEP programs, and further implementation of similar programs might improve PrEP use in the United States overall (*13*,*14*). Many states have passed legislation enabling pharmacists to independently initiate PrEP under collaborative practice agreements with physicians or a local public health department.

Support from FBOP leadership has helped to build the PrEP program, and provider trainings and patient education have helped drive patient assessments. In addition, allowing PrEP assessment without requiring patients to report a specific HIV risk behavior has helped to avoid the fear of stigma or disciplinary action for prohibited activities such as sexual activity and drug use. Provider training to avoid stigmatizing language and thereby encourage patient participation and PrEP uptake is essential for success (*15*,*16*).

Challenges to program implementation include adequate staffing (e.g., providers to prescribe medication, social workers to coordinate care upon release from custody). Allowing patients to self-refer for PrEP evaluation has helped to increase access despite staffing shortages. Although FBOP’s PrEP guidance was released in 2021, many facilities had to prioritize healthcare resources for the COVID-19 pandemic response at that time. A review of 46 studies suggests the COVID-19 pandemic disrupted continuity of PrEP care in a variety of settings (*17*). As FBOP transitions out of pandemic response and returns to routine care, integrating evaluation for PrEP as part of a holistic approach to preparing AICs for release back to their communities will enable improved access to care and harm reduction.

The first limitation of this study is that intake screening does not include all HIV risk factors (e.g., history of sex work) and does not assess an AIC’s predicted risk upon release from custody. Because screening is conducted at first intake and each time an AIC transfers to a new FBOP facility, risk factor data from intake screening might include duplicate records for some AICs.

FBOP will continue to educate employees and AICs to promote awareness of PrEP availability and reduce stigma. AIC education developed with the input of persons who are currently or previously incarcerated will help to ensure messaging is realistic and applicable to the incarcerated population. Efforts to address literacy barriers are being evaluated, such as verbally discussing harm reduction strategies to include PrEP and nasal naloxone at admission and orientation programs and during prerelease counseling. To assess progress and identify opportunities to increase the provision of PrEP, the FBOP is developing a dashboard to enable collection and evaluation of data at the national and institution level to include the number of AICs screened for PrEP eligibility, the number offered PrEP, and the percentage who accepted. In addition, FBOP will continue to involve pharmacists in HIV medication management and patient care activities, drawing on the successes and improved patient outcomes demonstrated to date.
